# Ethanol Sensing Properties and First Principles Study of Au Supported on Mesoporous ZnO Derived from Metal Organic Framework ZIF-8

**DOI:** 10.3390/s21134352

**Published:** 2021-06-25

**Authors:** Yanli Kang, Lu Zhang, Wenhao Wang, Feng Yu

**Affiliations:** 1 Key Laboratory for Green Processing of Chemical Engineering of Xinjiang Bingtuan, School of Chemistry and Chemical Engineering, Shihezi University, Shihezi 832003, China; 13040547128@163.com (Y.K.); 18299083557@163.com (W.W.); 2 School of Science, Harbin Institute of Technology, Shenzhen 518055, China; 18B925124@stu.hit.edu.cn

**Keywords:** gas sensor, zinc oxide, gold nanoparticles, ethanol, density functional theory

## Abstract

It is of great significance to develop ethanol sensors with high sensitivity and low detection temperature. Hence, we prepared Au-supported material on mesoporous ZnO composites derived from a metal-organic framework ZIF-8 for the detection of ethanol gas. The obtained Au/ZnO materials were characterized by X-ray diffraction (XRD), X-ray photoelectron spectroscopy (XPS), field emission scanning electron microscopy (SEM), field emission transmission electron microscopy (TEM) and nitrogen adsorption and desorption isotherms. The results showed that the Au/ZnO-1.0 sample maintains a three-dimensional (3D) dodecahedron structure with a larger specific surface area (22.79 m^2^ g^−1^) and has more oxygen vacancies. Because of the unique ZIF structure, abundant surface defects and the formation of Au-ZnO Schottky junctions, an Au/ZnO-1.0 sensor has a response factor of 37.74 for 100 ppm ethanol at 250 °C, which is about 6 times that of pure ZnO material. In addition, the Au/ZnO-1.0 sensor has good selectivity for ethanol. According to density functional theory (DFT) calculations, the adsorption energy of Au/ZnO for ethanol (−1.813 eV) is significantly greater than that of pure ZnO (−0.217 eV). Furthermore, the adsorption energy for ethanol is greater than that of other gases.

## 1. Introduction

In recent years research based on ethanol (C_2_H_5_OH) detection has received widespread attention due to its application in various fields such as detection of chemical plant leaks and the monitoring of drunk driving. C_2_H_5_OH, as a flammable volatile organic compound, is widely used in the food and beverage industry, medical industry and chemical industry [1]. When the air contains high concentrations of ethanol, it will cause safety accidents such as explosions [2], and cause health problems such as neurasthenia [3]. Although great progress has been made in the detection of ethanol, the present detection approaches still have shortcomings such as high operating temperature, low sensitivity and limited selectivity. Hence, more and more attention has been paid to the research of materials for high-performance ethanol detection. Modification of materials through the functionalization of precious metals is an effective strategy to improve the response of gas detection [4,5,6]. For example, doping Au in the ZnO/Zn_2_SnO_4_ structure could increase the gas sensitivity to H_2_S from 2.1 to 15.92 at 150 °C [7]. Moreover, the modification of Pd on ZnO can promote the dissociation of oxygen molecules to form oxygen anions, thereby increasing the sensor’s response to hydrogen from 0.11 to 123.53 [8].

The high catalytic activity of precious metals can promote the decomposition of gaseous substances, which is conducive to enhancing gas sensitivity [9,10]. In addition, the presence of precious metals on the surface of metal oxides increases the interaction between reducing gases and adsorbed oxygen on the surface [11]. However, precious metals have high surface energy and are easy to agglomerate, resulting in a decrease in the catalytic performance of the material. Therefore, it is necessary to develop convenient and reliable methods to precisely control the decoration and positioning of precious metal nanoparticles on the semiconductor surface. Metal-organic frameworks (MOFs) are often used as templates because of their higher specific surface area and ordered pore size (e.g., the pore size of zeolitic imidazolate framework (ZIF-8) is about 0.34 nm) [12]. MOFs can not only provide more active sites but also adjust the size of precious metal particles loaded on the surface of the material [13]. Encapsulating precious metal particles in the MOFs framework can effectively limit the size of precious metals to within a few nanometers and improve their dispersion and stability in semiconductors. As a result, the catalytic abilities of the precious metal particles in the system have been improved to a large extent [14].

In this work, with ZIF-8 as the precursor template, a three-dimensional (3D) ZnO structure supported by highly dispersed Au particles was prepared, which improved the sensing performance of C_2_H_5_OH effectively. The influence of different Au supported on the surface of ZnO on the detection of C_2_H_5_OH was discussed. The response factor (37.74 at 100 ppm) of the Au/ZnO (load: 1.79 wt.%) material derived from Au/ZIF-8 is six times that of pure ZnO material, and the response/recovery time is about 19/9 s. Finally, density functional theory (DFT) was used to estimate the adsorption energy of the Au/ZnO configuration for different gases. This shows that the sensor has great application potential in C_2_H_5_OH detection.

## 2. Materials and Methods

### 2.1. Chemicals

All chemicals are analytically pure and can be used directly without further purification. 1,2-Dimethylimidazole, sodium borohydride (NaBH, 98.0%) and hydrogen tetrachloroaurate trihydrate (HAuCl_4_·3H_2_O, 99.9%) were from Shanghai Adamas Reagent Co., Ltd, China. Zinc acetate dihydrate (C_4_H_6_O_4_Zn·2H_2_O, 99.0%) was purchased from Shanghai Titan Technology Co., Ltd., (Greagent), China. Methanol (CH_3_OH, 99.9%) and C_2_H_5_OH (99.7%) were from Tianjin Fuyu Fine Chemical Co., Ltd., China, and homemade deionized water.

### 2.2. Preparation of ZIF-8 Materials

The preparation of ZIF-8 adopted a simple co-precipitation method. We prepared clear solution 1 containing C_4_H_6_O_4_Zn·2H_2_O (3.81 g) and 200 mL CH_3_OH. 1,2-dimethylimidazole (9.25 g) and 200 mL of CH_3_OH were used to prepare clear solution 2. At room temperature, solution 2 was slowly added dropwise to solution 1, and then the mixed solution was continuously stirred for 24 h. During the stirring process, it was observed that white precipitates gradually appeared. Subsequently, the white precipitates (ZIF-8) were collected by centrifugation, washing with CH_3_OH 5 times, and vacuum drying at 60 °C for 12 h.

### 2.3. Preparation of Au Supported ZnO Materials

Prepared ZIF-8 powder (0.1 g) was put into CH_3_OH (5 mL) and agitated for 2 h to ensure uniform dispersion. We then added 1 mL of chloroauric acid aqueous solution (1 mg/mL) to the precursor solution and continued to stir for 1 h. In the above solution, an appropriate amount of NaBH (1.5 mg/mL) was added drop by drop and kept stirring for 2 h. The color of the solution (yellow) had a transition to purple in the process of NaBH solution addition, which indicated the transition from Au^3+^ to Au^0^. Finally, the purple suspension was washed with CH_3_OH and centrifuged for 3 cycles. The Au/ZIF-8 purple powder was obtained by drying under vacuum at 60 °C for 12 h. It was calcined at a rate of 10 °C/min to 500 °C for 2 h in a muffle furnace and then cooled naturally to room temperature to obtain the sample Au/ZnO-1.0. By adjusting the dropping amount of the chloroauric acid aqueous solution, ZnO with different amounts of Au modification was obtained. The obtained samples were named Au/ZnO-0.5 and Au/ZnO-1.5 by dropping 0.5 mL and 1.5 mL chloroauric acid aqueous solution, respectively. For comparative analysis, the ZIF-8 powder was directly calcined in a muffle furnace to obtain the comparative sample ZnO.

### 2.4. Gas Sensing Measurements

Metal oxide gas sensors are usually divided into two categories: indirect heating and direct heating, among which indirect gas sensors are used in this work. The synthesized material is mixed with a small amount of ethanol, and the mixed solution is coated on the ceramic tube substrate with a sample pen to form a thin and uniform coating of the sensing material. Then, in order to control the working temperature, the nickel-chromium coil is passed through the ceramic tube as a heater. Subsequently, the platinum wire and nickel-chromium heating wire pre-installed on the ceramic tube is welded to the base of the gas sensor. In order to improve the stability and repeatability of these sensor elements, the prepared sensor elements were aged at 170 °C for 24 h on an aging table. The gas sensor measurement is carried out using the CGS-8 intelligent gas sensor analysis system (Beijing Elite Technology Co., Ltd., Beijing, China) at an ambient temperature and relative humidity of 30 °C and 25–30%. In the process of testing, all the gases required in the test process adopt the liquid evaporation method. The response factor (*S*) of a material is defined as the ratio of the resistance (*R_a_*) of the gas-sensitive material in the air to the resistance (*R_g_*) in the target gas.

### 2.5. Material Characterization

X-ray diffraction (XRD, BRUCKER D8 ADVANCE, Berlin, Germany) provided a diffraction pattern of 10–80° diffraction range for analyzing the phase composition and crystal structure of the product. The Au content in different samples was determined by an inductively coupled plasma atomic emission spectrometer (ICP, Agilent ICPOES730, San-ta Clara, CA, USA). The morphology and structural characteristics of the samples were observed using a field emission scanning electron microscope (FE-SEM, SU-8020, Hitachi Corporation, Tokyo, Japan) and a field emission transmission electron microscope (TEM, JEM 2100F, JEOL Ltd., Tokyo, Japan). X-ray photoelectron spectroscopy (XPS, Thermo escalab 250Xi, Thermo Fisher Scientific, Waltham, MA, USA) was used to measure the chemical composition and valence state of the product elements. The specific surface area of the materials was measured by the physical adsorption instrument (ASAP 2460, Micromeritics instrument Ltd., Norcross, GA, USA) and the BET model, while the pore size distribution was calculated using the Barrett-Joyner-Halenda (BJH) method. An electron paramagnetic resonance (EPR, A300-10/12 Bruker, Berlin, Germany) test was used for quantitative analysis of oxygen vacancy.

## 3. Results and Discussion

### 3.1. Morphology and Structure of ZIF-8 Derived Au/ZnO Samples

**Figure 1:**
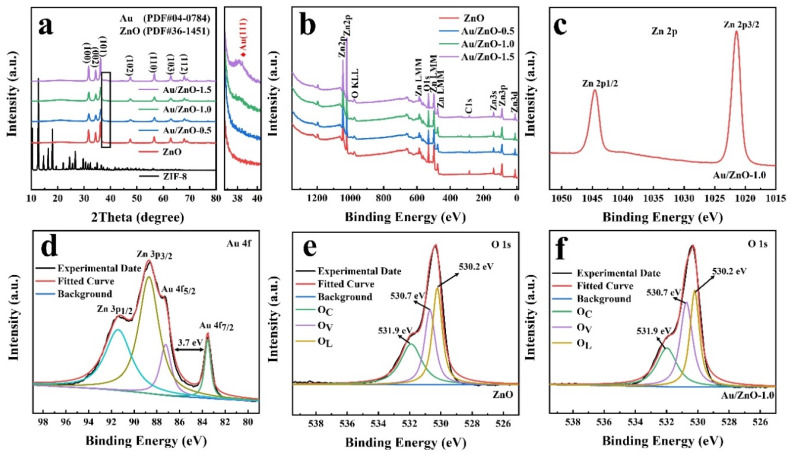
(**a**) XRD and (**b**) XPS spectra of ZnO and Au/ZnO samples; (**c**) XPS spectra of Zn 2p (Au/ZnO-1.0); (**d**) XPS spectra of Au 4f (Au/ZnO-1.0); and (**e**,**f**) XPS spectra of O 1s.

Figure 1a is the X-ray diffraction pattern of the prepared ZnO materials with different Au capacities. The profile of ZIF-8 is consistent with previous reports [15] proving that ZIF-8 was successfully synthesized. The diffraction peaks of pure ZnO at 2*θ* = 31.77°, 34.42°, 36.25°, 47.54°, 56.60°, 62.86° and 67.96°, which correspond to the reflection of (100), (002), (101), (102), (110), (103) and (112) crystal planes of wurtzite ZnO (PDF#36-1451). The figure on the right is the partially enlarged XRD diffraction pattern of ZnO loaded with different Au content, which shows that the peak shape of Au becomes more and more obvious as the Au load increases. When the added amount of Au reaches 1.0 mL, the XRD peak near 38.18° matches the (111) crystal plane of Au (PDF#04-0784), which proves that the synthesis of Au/ZnO composites is successful. When the Au added amount is 0.5 mL, no Au diffraction peaks are found, which is the reason for the low Au content or the uniform Au loading distribution [16]; this is also consistent with the ICP results (Table S1). The actual content of Au in the Au/ZnO-0.5, Au/ZnO-1.0 and Au/ZnO-1.5 samples are 0.92, 1.79 and 2.98 wt.%, respectively.

The full XPS spectrum of as-synthesized Au/ZnO samples shows the two main components of Zn and O, as shown in Figure 1b. In the Zn 2p spectra of the sample (Figure 1c), the corresponding peaks of Zn 2p_3/2_ and Zn 2p_1/2_ are at ∼1044.5 and ∼1021.3 eV, representing the oxidation state of Zn (II) [17]. The double peaks of Au 4f in Figure 1d are located at 87.2 eV and 83.5 eV, corresponding to the peaks of Au 4f_5/2_ and 4f_7/2_, respectively. The gap between Au 4f_7/2_ and Au 4f_5/2_ is consistent with the standard value (3.7 eV) of spin-orbit doublet separation, which belongs to the Au^0^ characteristic, proving that NaBH successfully converted Au^3+^ into Au^0^ [18,19,20]. Compared with the binding energy of pure metal Au 4f_7/2_ (84.0 eV) and 4f_5/2_ (87.7 eV), the reason why the binding energy of Au 4f in Au/ZnO materials decreased is the strong interaction between Au and ZnO [21,22,23]. Since the work function of Au (5.1 eV) [24] is higher than that of ZnO (4.45 eV) [16], electrons are transferred from ZnO to Au nanoparticles when Au nanoparticles contact ZnO. Zn 3p_1/2_ and Zn 3p_3/2_ are two interference peaks among the four peaks. Generally speaking, oxygen vacancies and chemisorbed oxygen species play an important role in gas sensing performance. Hence, we have performed deconvolution on the O 1s peaks of ZnO and Au/ZnO-1.0 materials (Figure 1e,f). The O 1s peak of pure ZnO can be divided into three peaks: 531.9 eV (O_C_), 530.7 eV (O_V_) and 530.2 eV (O_L_). The O_L_ peak with the lowest binding energy comes from the lattice oxygen on the hexagonal wurtzite structure, and the O_V_ peak represents the oxygen vacancies in the ZnO and Zn-OH matrix. The O_C_ peak with the highest binding energy usually represents the weaker oxygen adsorption on the surface of the ZnO material, such as H_2_O or O_2_ [25]. For the Au/ZnO-1.0 sample, it can be seen from Figure 1e that there is no obvious shift in the peak positions of O_C_, O_V_ and O_L_. The difference is the O_V_ peak area ratio of Au/ZnO is about 38.99% higher than that of pure ZnO (30.96%), which is consistent with the result of EPR (Figure S1). The modification of Au nanoparticles can increase the O_V_ content in ZnO materials, which can be attributed to the “spillover effect” of precious metals [26,27]. The increase of O_V_ can generate more free electrons, which will enhance the adsorption of surface gas, thus improving the gas-sensitive property of the materials [28,29].

**Figure 2:**
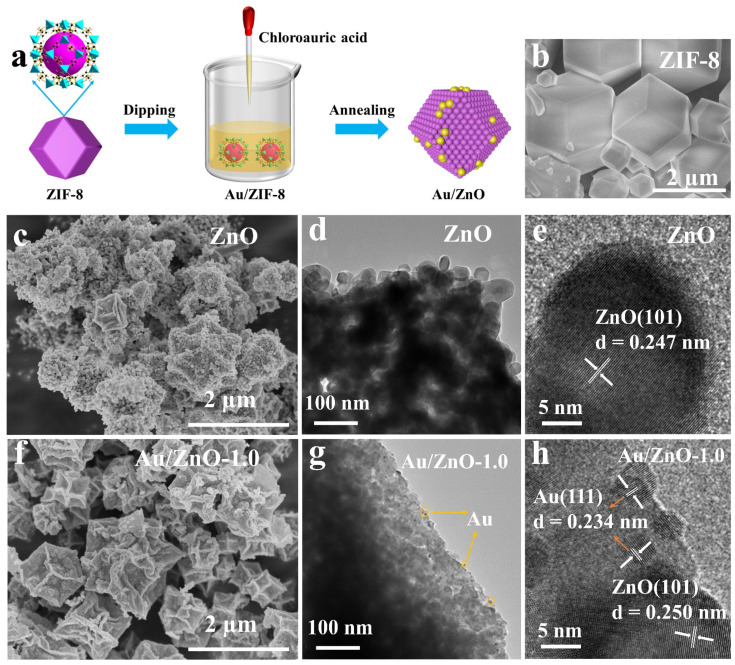
(**a**) Schematic diagram of preparation of Au/ZnO materials; SEM images of (**b**) ZIF-8, (**c**) ZnO and (**f**) Au/ZnO-1.0; TEM and HRTEM images of (**d**,**e**) ZnO and (**g**,**h**) Au/ZnO-1.0.

The ZIF-8 template (Figure 2b) is converted into ZnO powder after being calcined above 500 °C. The organic framework of ZIF-8 is decomposed during the firing process to produce a porous structure. The Zn ions near the surface of the ZIF-8 frame are directly oxidized to ZnO in the early stage of firing. In the later stage of firing, the diffusion rate of internal Zn ions and external oxygen determines the oxidation of internal Zn ions. Since the rate of outward diffusion of Zn ions is greater than that of inward diffusion of O ions, the internal voids are generated by the Kirkendall effect [30,31,32], as shown in Figure 2c. It can be clearly seen that the structure of ZIF-8 collapsed during the calcination process, and the obtained pure ZnO mainly presents a particle agglomeration structure with a small amount of dodecahedral structure. The morphologies of Au/ZnO samples derived from Au/ZIF-8 are shown in Figure 2f and Figure S2. With the loading of Au, the morphologies of Au/ZnO materials maintain the original dodecahedron structure of ZIF-8, and no obvious collapse occurred. This may be because Au ions dispersed in the pores of the metal-organic framework, which increases the stability of the ZIF-8 structure during the calcination process. In addition, ZIF-8 is a porous material with high porosity [33]. The 3D Au/ZnO materials prepared with ZIF-8 as a template also have a larger porosity, which is conducive to gas diffusion and increases the gas-sensitive reaction. Lou’s team [34] explored the gas-sensing mechanism of the dual effects of surface reaction control and gas diffusion control of SnO_2_ microspheres. When the reaction temperature is greater than 180 °C and less than 260 °C, the gas-sensing performance is dually affected by surface reaction control and gas diffusion control. When the temperature is greater than 260 °C, the gas sensitivity is mainly controlled by gas diffusion. 

The prepared Au/ZnO materials were studied by TEM and HRTEM to gain a deeper understanding of their structural characteristics (Figure 2 and Figure S3). It can be found that the more Au is loaded, the more Au particles distributed at the edge of ZnO material from Figure 2g. This indicates that Au particles have been successfully modified on the surface of ZnO. The HRTEM image (Figure 2e) of the ZnO material shows that it has the lattice fringe (0.247 nm) related to the (101) crystal plane of ZnO. Furthermore, as can be seen from Figure 2h, the lattice spacing of Au/ZnO contains two types: 0.250 nm and 0.234 nm, which correspond to the (101) crystal plane of ZnO and the (111) crystal plane of Au, respectively. The results of Figure 2g,h confirm that the reduced Au ions are uniformly dispersed on the surface of ZnO and exist in a zero-valence state. The EDS element map is shown in Figure S2g,h. These 3D dodecahedron structures are composed of Zn, O and Au, which further proves that Au elements are evenly distributed in the ZnO structure.

**Figure 3:**
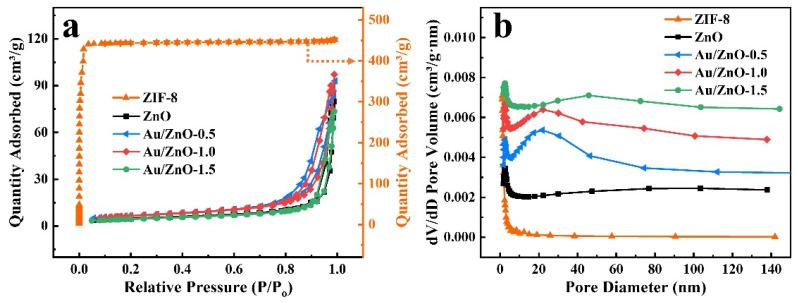
(**a**) N_2_ adsorption-desorption isotherms and (**b**) BJH pore size distribution of ZnO, Au/ZnO-0.5, Au/ZnO-1.0 and Au/ZnO-1.5 samples (each line is shifted upward by 0.0005 cm^3^/(g nm) in (**b**)).

The N_2_ adsorption-desorption isotherms (Figure 3) of ZIF-8, pure ZnO and Au/ZnO have been measured to analyze the specific surface area and pore structure of the materials. ZIF-8 has a high specific surface area (1479.70 m^2^ g^−1^), which is used as a template to provide more active sites during the reaction and enhance the gas adsorption capacity. Both ZnO and Au/ZnO show type IV isotherms with hysteresis loops, indicating that there are abundant mesoporous structures in Au/ZnO materials. The specific surface areas of ZnO, Au/ZnO-0.5, Au/ZnO-1.0 and Au/ZnO-1.5 estimated by the BET model are 16.79, 23.13, 22.97 and 15.65 m^2^ g^−1^, respectively. This result indicates that a proper amount of Au nanoparticles can increase the specific surface area of the material and the number of surface-active sites effectively. Secondly, the corresponding pore size distributions of ZnO, Au/ZnO-0.5, Au/ZnO-1.0 and Au/ZnO-1.5 are 5.67, 9.96, 7.14 and 5.37 nm. Larger pore sizes are more conducive to gas diffusion inside the material to improve the gas sensitivity of the material.

### 3.2. Au/ZnO Samples for C_2_H_5_OH Sensing Performance Testing and Analysis

**Figure 4:**
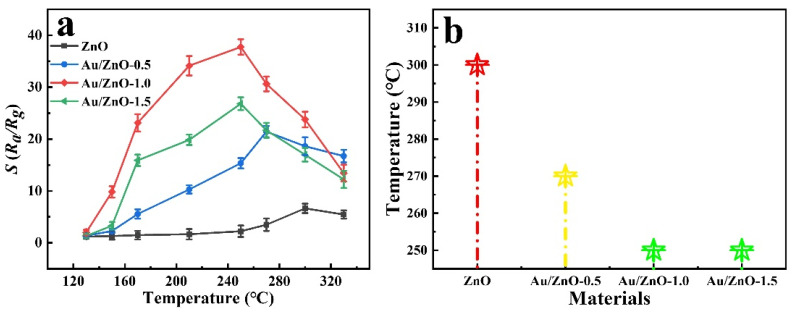
(**a**) ZnO, Au/ZnO-0.5, Au/ZnO-1.0 and Au/ZnO-1.5 working temperature and gas response diagram (100 ppm C_2_H_5_OH); (**b**) the best working temperature comparison diagram.

The gas response of a semiconductor sensor usually depends on the operating temperature of the sensor, which determines the surface state, gas adsorption/desorption and chemical reactions on the surface [3,35]. In a proper range, the increase in the working temperature is beneficial for the adsorption of oxygen and target gas on the surface of the materials. When the temperature is too high, the rapid dissociation and desorption of the surface gas will be accelerated. Although the resistance of general semiconductors at room temperature exceeds 1000 MΩ [36], the resistance of the Au/ZnO materials prepared in this article at room temperature also exceeds the maximum measurement range (500 MΩ). However, the resistance of the materials can be reduced by increasing the operating temperature. In addition, at temperatures below 130 °C, the prepared Au/ZnO and ZnO sensors have poor gas sensitivity. Therefore, the optimal temperature condition was determined by adjusting the operating temperature (130–330 °C) and observing the response of Au/ZnO and ZnO sensors to 100 ppm C_2_H_5_OH. It can be seen that the response of the prepared sensors becomes more intense with the increase of the working temperature. When the temperature reaches a certain value, the gas-sensing response begins to decrease as the temperature continues to increase (Figure 4a). When the response of the sensor is the highest, the corresponding temperature is the best working temperature for gas testing. As shown in Figure 4b, the optimal working temperature of the sensors based on Au/ZnO-1.0 and Au/ZnO-1.5 is reduced to 250 °C, and the response factor to 100 ppm C_2_H_5_OH increased to 37.74 and 26.81, which are almost six times and four times that of ZnO samples. This result shows that the addition of Au reduces the working temperature and improves the gas sensitivity. With the increase of the Au load, the gas sensitivity first increases and then decreases, when the load is 1.79 wt.% (data come from ICP) the gas-sensitive response is the largest. Therefore, the optimal load of Au is 1.79 wt.%, and 250 °C is the best working temperature for subsequent tests.

**Figure 5:**
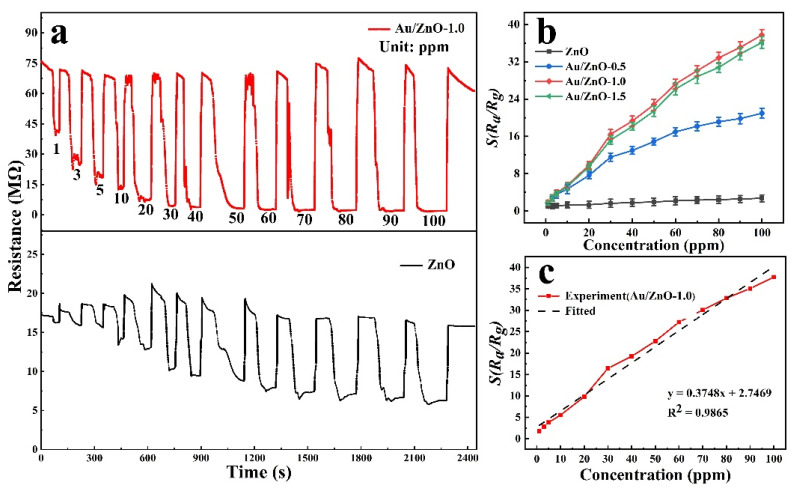
(**a**) Dynamic response and (**b**) concentration linearity curve of ZnO, Au/ZnO-0.5, Au/ZnO-1.0 and Au/ZnO-1.5 at 250 °C (C_2_H_5_OH: 1–100 ppm); (**c**) Au/ZnO-1.0 experimental data linear fitting curve.

The response of the Au/ZnO-1.0 sensor to 100 ppm C_2_H_5_OH is significantly higher than that of the sensor based on the ZnO in Table 1. In addition, the best working temperature of the Au/ZnO-1.0 sensor is 250 °C, which is lower than most reported best working temperatures. Figure 5a,b shows the characteristic curves of different C_2_H_5_OH concentrations (1–100 ppm) for pure ZnO and Au/ZnO-1.0 at 250 °C. The dynamic curves of other samples are shown in Figure S4. It can be seen clearly that the response of the four sensors increases with the gradual increase of the C_2_H_5_OH concentration. Furthermore, at different C_2_H_5_OH concentrations, the response of Au/ZnO is much higher than that of pure ZnO. This is because the catalysis of Au increases the oxidation rate of C_2_H_5_OH gas. The response of the Au/ZnO-1.0 sensor is higher than that of other sensors, with a response factor of 37.74 to 100 ppm C_2_H_5_OH. This is especially true at a low concentration of 1 ppm, with a response factor of 1.81 to C_2_H_5_OH, indicating that the Au/ZnO-1.0 sensor has a lower detection limit. Figure 5c is the linear fitting curve of the concentration at 250 °C. It can be seen that the Au/ZnO-1.0 sensor shows an approximately linear change in the C_2_H_5_OH concentration range of 1–100 ppm. The data fitting equation is as follows: y=0.3748 x+2.7469, and the correlation coefficient *R*^2^ is 0.9865. According to the slope of the linear fitting curve, the sensitivity of Au/ZnO-1.0 is 0.3748 ppm^−1^ in the range of 1 to 100 ppm. A low detection limit is important for providing early warning at the beginning of a leak.

**Figure 6:**
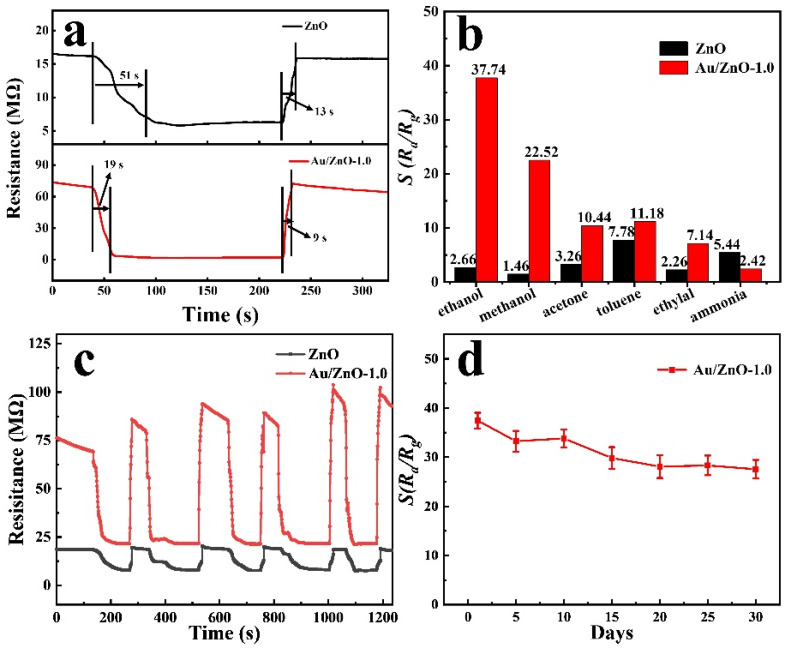
(**a**) The diagram of ZnO and Au/ZnO-1.0 response/recovery time; (**b**) ZnO and Au/ZnO-1.0 gas sensitivity response to other gases (100 ppm); (**c**) 5-cycle dynamic response-recovery curve; (**d**) Au/ZnO-1.0 sensor long-term stability test (C_2_H_5_OH: 100 ppm, n = 3, temperature: 250 °C).

The response (*T_Res_*)/recovery time (*T_Rec_*) of the sensor is also one of the important factors in the evaluation of gas performance. The *T_Res_*/*T_Rec_* is defined as the time required for the sensor element to reach 90% response or return to equilibrium when exposed to the target gas or air. A single cycle response of ZnO and Au/ZnO-1.0 sensor is shown in Figure 6a. The *T_Res_*/*T_Rec_* of Au/ZnO-1.0 is about 19/9 s, which is faster than that of a pure ZnO sensor (51/13 s). This shows that the Au-supported ZnO sensor has unique response-recovery characteristics. Good selectivity for a particular gas is a key parameter of gas sensors. In order to evaluate the selectivity of the sensor to C_2_H_5_OH at 250 °C, the response of ZnO and Au/ZnO-1.0 sensors to 100 ppm C_2_H_5_OH, CH_3_OH, acetone, toluene, acetaldehyde and ammonia are shown in Figure 6b. The results show that the response factor of the Au/ZnO-1.0 to C_2_H_5_OH gas is higher than that of other gases, which manifests that the Au/ZnO-1.0 has high selectivity to C_2_H_5_OH. In addition, the repeatability of ZnO and Au/ZnO materials to 100 ppm C_2_H_5_OH was also measured (Figure 6c and Figure S5). After five cycles of C_2_H_5_OH intake and diffusion, the ZnO and Au/ZnO sensors can still recover the initial resistance, indicating that the sensors have good repeatability. Furthermore, the response factor of the Au/ZnO-1.0 sensor to 100 ppm C_2_H_5_OH has very little downward trend (Figure 6d) after a one-month test period within the error range (n = 3, each data point is tested three times) showing their good reversibility and stability.

### 3.3. Gas Sensing Mechanism of the Au/ZnO Sensors

**Figure 7:**
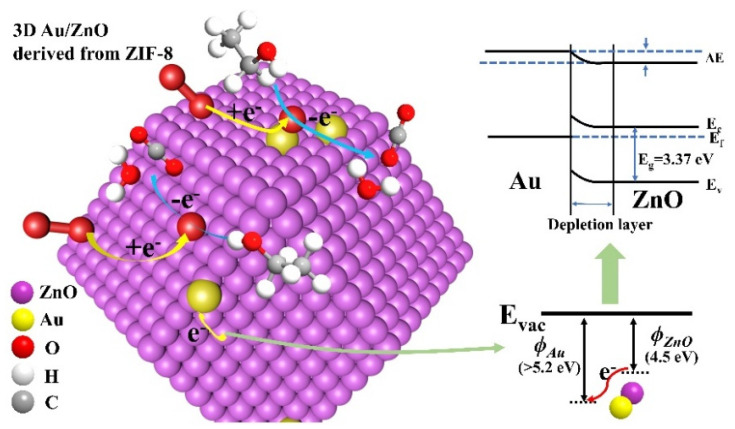
Sensing mechanism in the Au/ZnO gas sensor derived from ZIF-8.

The sensing mechanism of Au/ZnO materials can generally be explained by the surface adsorption oxygen model. Because the work function of Au (*ϕ* > 5.2 eV) is higher than that of ZnO (4.5 eV) [16,45], the electrons in ZnO move to Au, causing the electron concentration in Au particles to increase. When the Au/ZnO sensors are exposed to air, oxygen will be adsorbed on the Au/ZnO surface. Since electrons accumulate in Au particles, oxygen is more likely to contact Au particles and capture electrons to form negative oxygen ions (O_2_^−^, O^−^, O^2−^), which leads to an increase in the initial resistance. When the reducing gas (C_2_H_5_OH in our case) is injected into the test chamber, C_2_H_5_OH can react with oxygen ions, losing two hydrogen atoms to generate acetaldehyde molecules at first. Then the decomposed acetaldehyde will further react with the adsorbed oxygen ions on the surface to form acetic acid, which is then decomposed into carbon dioxide and water molecules. During this adsorption reaction, the lost electrons of Au/ZnO will be released back into the material to reduce the resistance. This paper presents the sensing mechanism diagram of the 3D Au/ZnO sensor derived from ZIF-8 (Figure 7).

The improvement of the sensing performance of 3D Au/ZnO derived from Au/ZIF-8 can be explained by three factors, namely the size effect, the composition design of the sensing material, and the structure design. First, the size effect of Au nanoparticles in the 3D Au/ZnO sensor derived from ZIF-8 should be considered [14]. When the size is reduced to the nanoscale, the catalytic performance of precious metals can be improved effectively [46,47]. In particular, Au nanoparticles can disperse more uniformly, and their size is easier to control under the protection of the ZIF-8 shell layer so that they have a better detection capability for C_2_H_5_OH. While Au nanoparticles supported on the surface without using the ZIF structure as a template are prone to agglomeration due to their high surface energy, enervating catalytic performance [48,49]. Secondly, when introducing precious metals into semiconductor-based resistance sensing devices, the mechanisms of “electronic sensitization” and “chemical sensitization” should be considered [50,51,52]. Electron sensitization occurs because the electrons in ZnO transfer to Au when Au nanoparticles are in contact with ZnO, which expands the depletion zone of ZnO. The enlarged depletion region increases the initial barrier, which in turn increases the initial resistance value of ZnO and enhances the response strength. Chemical sensitization is attributed to the catalysis of oxygen separation. The addition of Au nanoparticles helps to reduce the activation energy of oxygen separation into oxygen ions, so more oxygen molecules can be adsorbed on the surface of ZnO to form oxygen anions, which is also known as the “spillover effect”. The generated oxygen ions can react with more C_2_H_5_OH gas to enhance the gas response. In addition, the high specific surface area of the 3D dodecahedron structure provides abundant active sites and the high porosity affords more channels for gas diffusion, which lay a good foundation for high-performance sensors.

### 3.4. Adsorption Characteristics of the Au/ZnO Sensors

**Figure 8:**
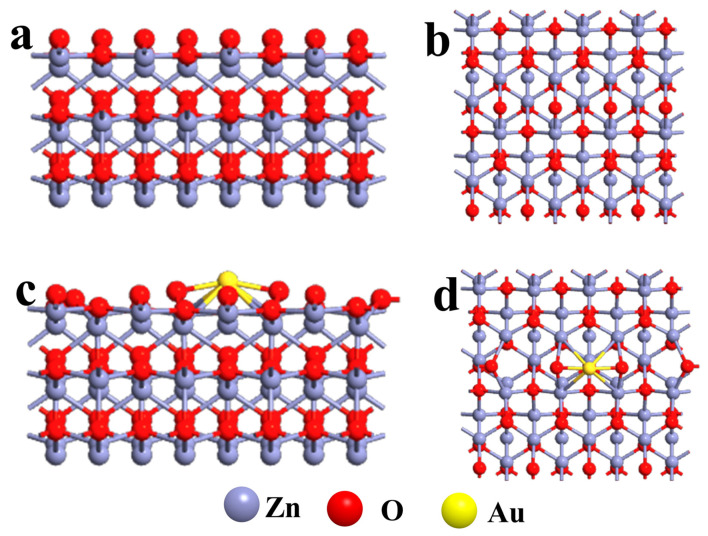
Optimized geometry of (**a**,**b**) ZnO and (**c**,**d**) Au/ZnO (side view on the left, top view on the right).

DFTs have been extensively utilized to analyze the sensing properties of different materials and great successes have been achieved in various fields [5,53,54]. For example, Monrudee Liangruksa’s team applied DFT to study the sensing properties of palladium-modified ZnO nanofilms to target gases (i.e., hydrogen (H_2_) and C_2_H_5_OH). The calculation results showed that, compared with bare ZnO, modified ZnO had a significant promotion effect on the reaction of H_2_ and C_2_H_5_OH. The gas sensitivity of the sensor for H_2_ and C_2_H_5_OH detection is mainly attributed to the surface modification and its resulting electron transfer mechanism, DOS, band gap and gas adsorption [55]. Hence, in our work, the first principles analysis of Au/ZnO structures based on DFT and linear combinations of atomic orbitals (LCAO) basis set was performed using the Atomistix Toolkit (ATK) to investigate the adsorption properties of the surface of the materials. It is well known that the first-principles calculations used by the DFT are usually based on absolute zero. With regard to direct band-gap semiconductors, the random motion of atoms under room temperature can be considered a negligible interference. Therefore, absolute zero is used in the first principles calculation of the Au/ZnO structure. Based on XRD and HRTEM analyses, the crystal plane model of ZnO (101) loaded with Au was constructed. The Au/ZnO configuration contains 97 atoms (48 Zn atoms, 48 O atoms and 1 Au atom). The Au/ZnO structure is periodic in the x and y planes, and there is a vacuum layer of 10 Å on the z-axis, in order to avoid imaginary interactions between adjacent atoms. In all calculations, the bottom three layers of the Au/ZnO configuration (a total of six atomic layers) remain fixed. The generalized gradient approximation (GGA) of Perdew-Burke-Ernzerh (PBE) represents the exchange-correlation effect of electrons in the DFT calculation. Density mesh cut-off was set to 125 Hartree, and the first Brillouin zones are sampled using 2 × 3 × 1 k-points in the x, y and z directions, respectively. The LBFGS algorithm was used to relax the positions of all atoms until the force on each atom was less than 0.05 eV/Å. The geometry of the configuration was optimized (Figure 8).

In order to simulate the adsorption characteristics of oxygen molecules on the surface of the material, an oxygen molecule was placed on the optimized ZnO and Au/ZnO configurations, about 3 Å from the surface of the configuration, and the configurations were optimized. By continuously adjusting the position of the oxygen molecules, the geometric configuration with the lowest system energy was obtained. The adsorption energies (*E_ads_*) of ZnO and Au/ZnO configurations for oxygen were calculated according to the energy of the system. The adsorption energies of the gas molecules on the ZnO and Au/ZnO system are obtained by:

$$E_{\mathrm{ads}}{=E}_{\left(\mathrm{adsorbate}/\mathrm{slab}\right)}-{\;(E}_{\mathrm{slab}}{+E}_{\mathrm{adsorbate}})$$

**Figure 9:**
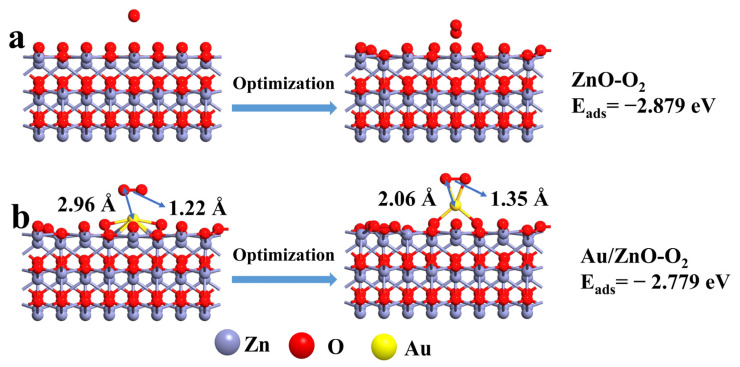
The optimized adsorption model of oxygen on the surface of (**a**) ZnO and (**b**) Au/ZnO.

Herein, *E_(adsorbate/slab)_* is the total energy of the system interacting with the surface configuration and adsorbate, *E_(slab)_* and *E_(adsorbate)_* are the energy of surface configuration and adsorbate respectively. Therefore, negative energy indicates that the adsorption process is exothermic. The larger the absolute value of the calculated adsorption energy is, the stronger the adsorption is [56]. It can be seen from Figure 9 that the adsorption energy of ZnO for oxygen is −2.879 eV, which is slightly larger than that of Au/ZnO for oxygen (−2.779 eV). Moreover, it can be seen that the oxygen molecules are obviously close to the configuration surface, and the distance between the oxygen molecules and the Au/ZnO configuration surface is shortened from 2.96 Å to 2.06 Å. The elongation of the bond length can be explained by the fact that the oxygen molecules which are adsorbed on the surface of the configuration will extract the surface charge of the Au/ZnO. Furthermore, the electron concentration in the oxygen molecule increased and the O-O bond weakened.

**Figure 10:**
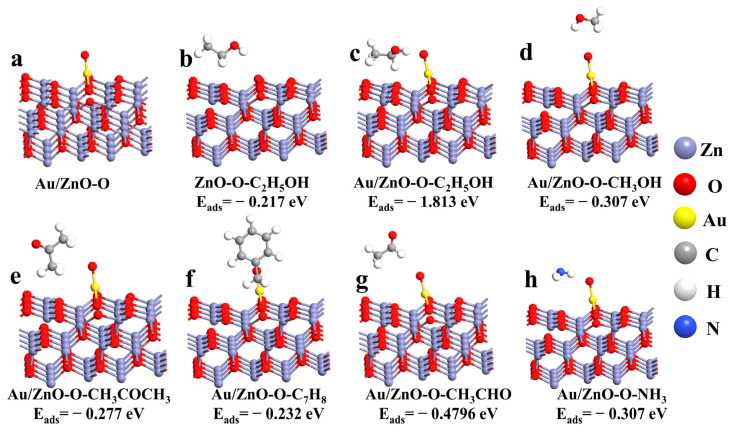
(**a**) The optimized adsorption model of Au/ZnO-O; The optimized adsorption model of different gases on the surface of ZnO-O (**b**: C_2_H_5_OH) and Au/ZnO-O (**c**: C_2_H_5_OH; **d**: CH_3_OH; **e**: CH_3_COCH_3_; **f**: C_7_H_8_; **g**: CH_3_CHO; **h**: NH_3_).

According to the above simulation analysis, it can be seen that the oxygen molecule has a tendency of cracking during the adsorption process. However, our working temperature was 250 °C in the actual gas test. High temperature can reduce the activation energy of gas adsorption and provide energy for the cracking of oxygen molecules. Therefore, we simulated the adsorption of a single oxygen atom on the Au/ZnO configuration surface (Figure 10a) from experimental considerations. The optimized Au/ZnO-O model was used to explore the interaction between different gases and adsorbed oxygen on the surface, and various gas molecules were placed about 3 Å away from the Au/ZnO configuration surface. Then the structure was optimized to obtain the lowest energy configuration of the system. Figure 10b–h is a front view of the structure of Au/ZnO-O adsorbed by different gases. It can be seen from the comparison of adsorption energy that the adsorption capacity of the Au/ZnO system to C_2_H_5_OH is significantly stronger than that of the ZnO system. Furthermore, the adsorption intensity of the Au/ZnO system to CH_3_OH, acetone, toluene, acetaldehyde and ammonia is less than that of the system to C_2_H_5_OH. Hence, DFT calculations prove that Au-supported ZnO materials can improve the adsorption of ZnO to C_2_H_5_OH, enhancing the gas-sensing response. Moreover, through the comparison of different gas adsorption energies, it proves that Au/ZnO materials have good selectivity to C_2_H_5_OH. 

## 4. Conclusions

In conclusion, Au nanoparticles were successfully supported on ZIF-8 derived ZnO. The gas-sensing characteristics of Au/ZnO materials were verified by DFT calculation. When the Au load is 1.79 wt.%, the response of the obtained sensor (Au/ZnO-1.0) to C_2_H_5_OH is improved significantly. The response of the Au/ZnO-1.0 sensor to 100 ppm C_2_H_5_OH at 250 °C is 37.74, which is about 6 times that of pure ZnO material. The sensor also has a faster response/recovery time, about 19/9 s. In addition, the gas sensor has good selectivity, repeatability and stability to C_2_H_5_OH. The DFT calculation shows that the adsorption energies of ZnO and Au/ZnO for oxygen are −2.879 eV and −2.779 eV, and the O-O bond has been elongated. The adsorption energies of adsorbed oxygen on the ZnO and Au/ZnO surface to C_2_H_5_OH are −0.217 and −1.813 eV, and the adsorption strength of Au/ZnO to C_2_H_5_OH is significantly higher than that of several gases. In summary, the loading of Au further enhances the gas adsorption on the surface of ZnO and reduces the activation energy for gas adsorption and reaction. Simultaneously, the size of Au nanoparticles can be adjusted to a certain extent under the protection of the ZIF-8 shell layer, and they are evenly dispersed on the three-dimensional ZnO framework, thereby effectively enhancing the sensing performance of C_2_H_5_OH gas.

## Figures and Tables

**Table 1:** The gas sensitivity of ZnO-based sensors for the detection of C_2_H_5_OH.

Materials	Concentration (ppm)	Temperature (°C)	Response (*R_a_/R_g_*)	LOD (ppm)	Ref.
CuO/ZnO nanowires	100	300	28	1.06	[37]
Au,Cl-ZnO nanoparticles	100	220	19.64	19.64	[38]
T-ZnO/ZnFe_2_O_4_ Tetrapods	100	300	13.95	1	[39]
Mg doped ZnO	5	300	80	1	[40]
Ag/ZnO nanorods	100	360	36.6	50	[41]
K-doped ZnO nanorods	100	300	45.2	2	[42]
Ag-ZnO	50	325	32.5	50	[43]
ZnO nanorods	100	300	44.9	10	[44]
Au/ZnO	100	250	37.74	1	This work

## Supplementary Materials

The following are available online at https://www.mdpi.com/article/10.3390/s21134352/s1, Figure S1: SEM images of (a) Au/ZnO-0.5, (b) Au/ZnO-1.5. Figure S2: (a,b) TEM images of Au/ZnO-0.5 and Au/ZnO-1.5, (c,d) Mapping spectrum of Au/ZnO-1.0. Figure S3: Dynamic response graph of (a) Au/ZnO-1.5 and (b) Au/ZnO-0.5 samples to C_2_H_5_OH (1–100 ppm). Figure S4: 5-cycle dynamic response-recovery curve of (a) Au/ZnO-0.5 and (b) Au/ZnO-1.5 samples to C_2_H_5_OH (100 ppm). Table S1: Au element content in Au/ZnO samples (ICP).

## References

[1] Li B., Liu J., Liu Q., Chen R., Zhang H., Yu J., Song D., Li J., Zhang M., Wang J. (2019). Core-shell structure of ZnO/Co_3_O_4_ composites derived from bimetallic-organic frameworks with superior sensing performance for ethanol gas. Appl. Surf. Sci..

[2] Cao P., Yang Z., Navale S.T., Han S., Liu X., Liu W., Lu Y., Stadler F.J., Zhu D. (2019). Ethanol sensing behavior of Pd-nanoparticles decorated ZnO-nanorod based chemiresistive gas sensors. Sens. Actuators B.

[3] Zhang K., Qin S., Tang P., Feng Y., Li D. (2020). Ultra-sensitive ethanol gas sensors based on nanosheet-assembled hierarchical ZnO-In_2_O_3_ heterostructures. J. Hazard. Mater..

[4] Kang Y., Yu F., Zhang L., Wang W., Chen L., Li Y. (2021). Review of ZnO-based nanomaterials in gas sensors. Solid State Ion..

[5] Yan Z., Tao C., Bai Y., Liu S. (2021). Adsorption of nitrogen based gas molecules on noble metal functionalized carbon nitride nanosheets: A theoretical investigation. Comput. Theor. Chem..

[6] Chauhan P.S., Mishra A., Bhatt G., Bhattacharya S. (2021). Enhanced He gas detection by V_2_O_5_-noble metal (Au, Ag, and Pd) nanocomposite with temperature dependent n- to p-type transition. Mater. Sci. Semicond. Process..

[7] Zhang S., Liu Z., Zhang L., Chen J., Zhou Q., Zhang H., Nie L., Dong Z., Zhang Z.A., Wang Z. (2021). Construction of a low-temperature, highly sensitive H_2_S sensor based on surfaces and interfaces reaction triggered by Au-doped hierarchical structured composites. Chem. Phys. Lett..

[8] Cheng J., Hu D., Yao A., Gao Y., Asadi H. (2020). A computational study on the Pd-decorated ZnO nanocluster for H_2_ gas sensing: A comparison with experimental results. Phys. E Low-Dimens. Syst. Nanostruct..

[9] Hsueh T.-J., Wu S.-S. (2021). Highly sensitive Co_3_O_4_ nanoparticles/MEMS NO_2_ gas sensor with the adsorption of the Au nanoparticles. Sens. Actuators B Chem..

[10] Cui W., Kang X., Zhang X., Zheng Z., Cui X. (2019). Facile synthesis of porous cubic microstructure of Co_3_O_4_ from ZIF-67 pyrolysis and its Au doped structure for enhanced acetone gas-sensing. Phys. E Low-Dimens. Syst. Nanostruct..

[11] Kohl D. (1990). The role of noble metals in the chemistry of solid-state gas sensors. Sens. Actuators B Chem..

[12] Koo W.T., Qiao S., Ogata A.F., Jha G., Jang J.S., Chen V.T., Kim I.D., Penner R.M. (2017). Accelerating palladium nanowire H_2_ sensors using engineered nanofiltration. ACS Nano.

[13] Hsueh T.-J., Peng C.-H., Chen W.-S. (2020). A transparent ZnO nanowire MEMS gas sensor prepared by an ITO micro-heater. Sens. Actuators B Chem..

[14] Zhou X., Lin X., Yang S., Zhu S., Chen X., Dong B., Bai X., Wen X., Geyu L., Song H. (2020). Highly dispersed Metal–Organic-Framework-Derived Pt nanoparticles on three-dimensional macroporous ZnO for trace-level H_2_S sensing. Sens. Actuators B Chem..

[15] Wang J., Han G., Wang L., Du L., Chen G., Gao Y., Ma Y., Du C., Cheng X., Zuo P. (2018). ZIF-8 with ferrocene encapsulated: A promising precursor to single-atom Fe embedded nitrogen-doped carbon as highly efficient catalyst for oxygen electroreduction. Small.

[16] Zhang B., Wang Y., Meng X., Zhang Z., Mu S. (2020). High response methane sensor based on Au-modified hierarchical porous nanosheets-assembled ZnO microspheres. Mater. Chem. Phys..

[17] Zhang N., Yan L., Lu Y., Fan Y., Guo S., Adimi S., Liu D., Ruan S. (2020). Metal-organic frameworks-derived hierarchical ZnO structures as efficient sensing materials for formaldehyde detection. Chin. Chem. Lett..

[18] Zhang L., Dong R., Zhu Z., Wang S. (2017). Au nanoparticles decorated ZnS hollow spheres for highly improved gas sensor performances. Sens. Actuators B Chem..

[19] Zhang J., Liu X., Wang L., Yang T., Guo X., Wu S., Zhang S., Wang S. (2011). A simple one-pot strategy for the synthesis of ternary reduced graphite oxide/SnO_2_/Au hybrid nanomaterials. Carbon.

[20] Zhang J., Liu X., Wu S., Xu M., Guo X., Wang S. (2010). Au nanoparticle-decorated porous SnO_2_ hollow spheres: A new model for a chemical sensor. J. Mater. Chem..

[21] Yu S., Zhang H., Chen C., Lin C. (2019). Investigation of humidity sensor based on Au modified ZnO nanosheets via hydrothermal method and first principle. Sens. Actuators B Chem..

[22] Li H., Chu S., Ma Q., Fang Y., Wang J., Che Q., Wang G., Yang P. (2019). Novel construction of morphology-tunable C-N/SnO_2_/ZnO/Au microspheres with ultrasensitivity and high selectivity for triethylamine under various temperature detections. ACS Appl. Mater. Interfaces.

[23] Yang T.H., Huang L.D., Harn Y.W., Lin C.C., Chang J.K., Wu C.I., Wu J.M. (2013). High density unaggregated Au nanoparticles on ZnO nanorod arrays function as efficient and recyclable photocatalysts for environmental purification. Small.

[24] Kumaravel V., Mathew S., Bartlett J., Pillai S.C. (2018). Photocatalytic hydrogen production using metal doped TiO_2_: A review of recent advances. Appl. Catal. B.

[25] Shingange K., Tshabalala Z.P., Ntwaeaborwa O.M., Motaung D.E., Mhlongo G.H. (2016). Highly selective NH_3_ gas sensor based on Au loaded ZnO nanostructures prepared using microwave-assisted method. J. Colloid Interface Sci..

[26] Xing R., Li Q., Lei X., Jian S., Song H. (2015). Au modified three-dimensional In_2_O_3_ inverse opals: Synthesis and improved performance for acetone sensing toward diagnosis of diabetes. Nanoscale.

[27] Yang S., Sun J., Xu L., Zhou Q., Chen X., Zhu S., Dong B., Lu G., Song H. (2020). Au@ZnO functionalized three–dimensional macroporous WO_3_: A application of selective H_2_S gas sensor for exhaled breath biomarker detection. Sens. Actuators B.

[28] Zhou X., Wang A., Wang Y., Bian L., Yang Z., Bian Y., Gong Y., Wu X., Han N., Chen Y. (2018). Crystal defect dependent gas sensing mechanism of the single ZnO nanowire sensors. ACS Sens..

[29] Wei W., Zhao J., Shi S., Lin H., Mao Z., Zhang F., Qu F. (2020). Boosting ppb-level triethylamine sensing of ZnO: Adjusting proportions of electron donor defects. J. Mater. Chem. C.

[30] Zhang R., Zhou T., Wang L., Zhang T. (2018). Metal-organic frameworks-derived hierarchical Co_3_O_4_ structures as efficient sensing materials for acetone detection. ACS Appl. Mater. Interfaces.

[31] Lu H.-B., Liao L., Li H., Wang D.-F., Tian Y., Li J.-C., Fu Q., Zhu B.-P., Wu Y. (2008). Hollow MgO nanotube arrays by using ZnO nanorods as templates. Eur. J. Inorg. Chem..

[32] Wang W., Dahl M., Yin Y. (2012). Hollow nanocrystals through the nanoscale kirkendall effect. Chem. Mater..

[33] Li W., Wu X., Liu H., Chen J., Tang W., Chen Y. (2015). Hierarchical hollow ZnO cubes constructed using self-sacrificial ZIF-8 frameworks and their enhanced benzene gas-sensing properties. New J. Chem..

[34] Wang X., Wang Y., Tian F., Liang H., Wang K., Zhao X., Lu Z., Jiang K., Yang L., Lou X. (2015). From the surface reaction control to gas-diffusion control: The synthesis of hierarchical porous SnO_2_ microspheres and their gas-sensing mechanism. J. Phys. Chem. C.

[35] Xiong H., Fu J., Li J., Ali R., Wang H., Liu Y., Su H., Li Y., Lau W.-M., Mahmood N. (2021). Strain-regulated sensing properties of α-Fe2O3 nano-cylinders with atomic carbon layers for ethanol detection. J. Mater. Sci. Technol..

[36] Yu Z., Gao J., Xu L., Liu T., Liu Y., Wang X., Suo H., Zhao C. (2020). Fabrication of lettuce-like ZnO gas sensor with enhanced H_2_S gas sensitivity. Crystals.

[37] Zhao S., Shen Y., Hao F., Kang C., Meng F. (2021). P-n junctions based on CuO-decorated ZnO nanowires for ethanol sensing application. Appl. Surf. Sci..

[38] Zhang Y., Zhao X., Hao W., Sun L., Cao E. (2020). Ethanol sensing characteristics of Au and Cl-comodified ZnO nanoparticles. Mater. Lett..

[39] Mei H., Zhou S., Lu M., Cheng L. (2020). Tetrapod-like ZnO/ZnFe_2_O_4_ based heterostructure for enhanced ethanol detection. J. Alloys Compd..

[40] Jaballah S., Benamara M., Dahman H., Ly A., Mir L. (2020). Effect of Mg-doping ZnO nanoparticles on detection of low ethanol concentrations. Mater. Chem. Phys..

[41] Wei Y., Wang X., Yi G., Zhou L., Cao J., Sun G., Chen Z., Bala H., Zhang Z. (2017). Hydrothermal synthesis of Ag modified ZnO nanorods and their enhanced ethanol-sensing properties. Mater. Sci. Semicond. Process..

[42] Saaedi A., Yousefi R. (2017). Improvement of gas-sensing performance of ZnO nanorods by group-I elements doping. J. Appl. Phys..

[43] Yousefi H.R., Hashemi B., Mirzaei A., Roshan H., Sheikhi M.H. (2020). Effect of Ag on the ZnO nanoparticles properties as an ethanol vapor sensor. Mater. Sci. Semicond. Process..

[44] Zhao S., Shen Y., Yan X., Zhou P., Yin Y., Lu R., Han C., Cui B., Wei D. (2019). Complex-surfactant-assisted hydrothermal synthesis of one-dimensional ZnO nanorods for high-performance ethanol gas sensor. Sens. Actuators B Chem..

[45] Sundaram K.B., Khan A. (1997). Work function determination of zinc oxide films. J. Vac. Sci. Technol..

[46] Lee J.-S., Katoch A., Kim J.-H., Kim S.S. (2016). Effect of Au nanoparticle size on the gas-sensing performance of p-CuO nanowires. Sens. Actuators B Chem..

[47] Zhang Y., Xu J., Xu P., Zhu Y., Chen X., Yu W. (2010). Decoration of ZnO nanowires with Pt nanoparticles and their improved gas sensing and photocatalytic performance. Nanotechnology.

[48] He L., Liu Y., Liu J., Xiong Y., Zheng J., Liu Y., Tang Z. (2013). Core-shell noble-metal@metal-organic-framework nanoparticles with highly selective sensing property. Angew. Chem..

[49] Fu F., Wang C., Wang Q., Martinez-Villacorta A.M., Escobar A., Chong H., Wang X., Moya S., Salmon L., Fouquet E. (2018). Highly selective and sharp volcano-type synergistic Ni_2_Pt@ZIF-8-catalyzed hydrogen evolution from ammonia borane hydrolysis. J. Am. Chem. Soc..

[50] Lee J., Jung Y., Sung S.-H., Lee G., Kim J., Seong J., Shim Y.-S., Jun S.C., Jeon S. (2021). High-performance gas sensor array for indoor air quality monitoring: The role of Au nanoparticles on WO_3_, SnO_2_, and NiO-based gas sensors. J. Mater. Chem. A.

[51] Rai P., Majhi S.M., Yu Y.-T., Lee J.-H. (2015). Noble metal@metal oxide semiconductor core@shell nano-architectures as a new platform for gas sensor applications. RSC Adv..

[52] Velmathi G., Mohan S., Henry R. (2015). Analysis of factors for improving functionality of tin oxide gas sensor. IETE Tech. Rev..

[53] Li J.-H., Wu J., Yu Y.-X. (2021). DFT exploration of sensor performances of two-dimensional WO_3_ to ten small gases in terms of work function and band gap changes and I-V responses. Appl. Surf. Sci..

[54] Fu J., Ali R., Mu C., Liu Y., Mahmood N., Lau W.-M., Jian X. (2021). Large-scale preparation of 2D VSe_2_ through a defect-engineering approach for efficient hydrogen evolution reaction. Chem. Eng. J..

[55] Liangruksa M., Sukpoonprom P., Junkaew A., Photaram W., Siriwong C. (2021). Gas sensing properties of palladium-modified zinc oxide nanofilms: A DFT study. Appl. Surf. Sci..

[56] Ni Z., Bao S., Gong X.-Q. (2020). A DFT study of the CO adsorption and oxidation at ZnO surfaces and its implication for CO detection. Chin. Chem. Lett..

