# Supplementary material for: Ethanol Sensing Properties and First Principles Study of Au Supported on Mesoporous ZnO Derived from Metal Organic Framework ZIF-8
Source: Sensors (Basel). 2021 Jun 25;21(13):4352. doi: 10.3390/s21134352 (PMC8272103; doi:10.3390/s21134352)
Supplement: Supplementary file 1 [file sensors-21-04352-s001.zip › Highlights.pdf]

Highlights:

- Using ZIF-8 as a template effectively improves dispersion of Au particles on the surface of ZnO.
- Ethanol response of Au/ZnO-1.0 is superior to methanol, toluene, acetone, acetaldehyde and ammonia.
- Density functional theory is employed to analyze the adsorption characteristics of Au/ZnO.
