# Supplementary material for: Ethanol Sensing Properties and First Principles Study of Au Supported on Mesoporous ZnO Derived from Metal Organic Framework ZIF-8
Source: Sensors (Basel). 2021 Jun 25;21(13):4352. doi: 10.3390/s21134352 (PMC8272103; doi:10.3390/s21134352)
Supplement: Supplementary file 1 [file sensors-21-04352-s001.zip › Supporting information.pdf]

Supporting information

Ethanol Sensing Properties and First Principles Study of Au Supported on Mesoporous ZnO Derived from Metal Organic Framework ZIF-8

Yanli Kang [1], Lu Zhang [2], Wenhao Wang [1] and Feng Yu [1,*]

[1] Key Laboratory for Green Processing of Chemical Engineering of Xinjiang Bingtuan, School of Chemistry and Chemical Engineering, Shihezi University, Shihezi 832003, P.R. China; 13040547128@163.com (Y.K.); 18299083557@163.com (W.W.)

[2] School of Science, Harbin Institute of Technology, Shenzhen 518055, P.R. China; 18B925124@stu.hit.edu.cn (L. Z.)

* Correspondence: yufeng05@mail.ipc.ac.cn (F.Y)

Table S1. Au element content in Au/ZnO samples (ICP).

Materials	Element	Content(wt.%)
ZnO	--	--
Au/ZnO-0.5	Au	0.9194
Au/ZnO-1.0	Au	1.7895
Au/ZnO-1.5	Au	2.9764

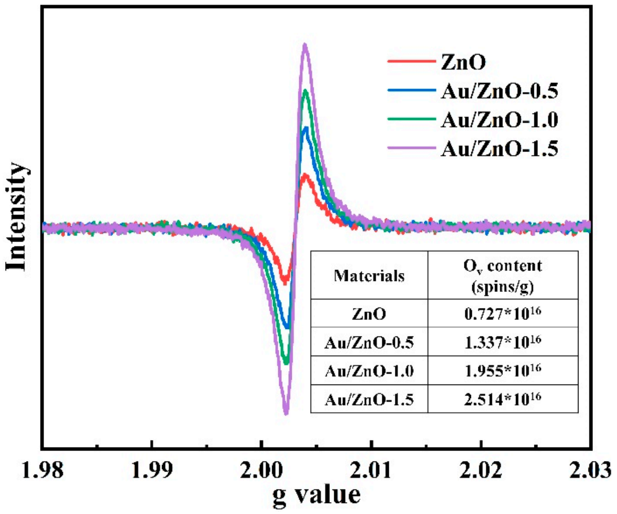

Figure S1. Quantitative analysis of oxygen vacancies in Au/ZnO.

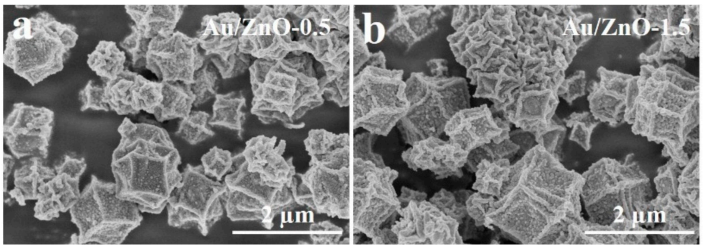

Figure S2. SEM images of (**a**) Au/ZnO-0.5, (**b**) Au/ZnO-1.5.

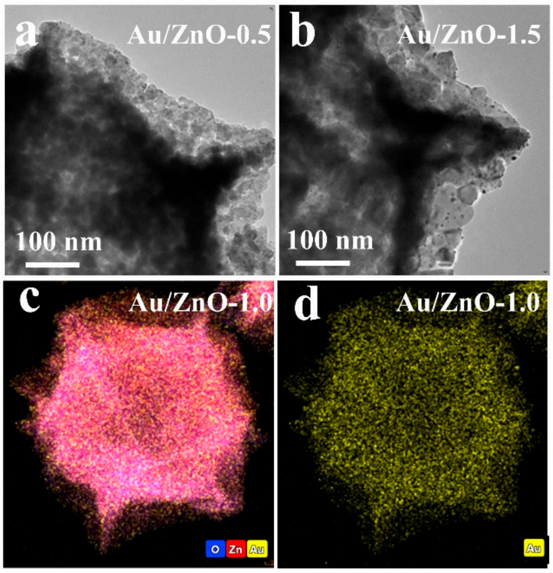

Figure S3. (**a**–**b**) TEM images of Au/ZnO-0.5 and Au/ZnO-1.5; (**c**–**d**) Mapping spectrum of Au/ZnO-1.0.

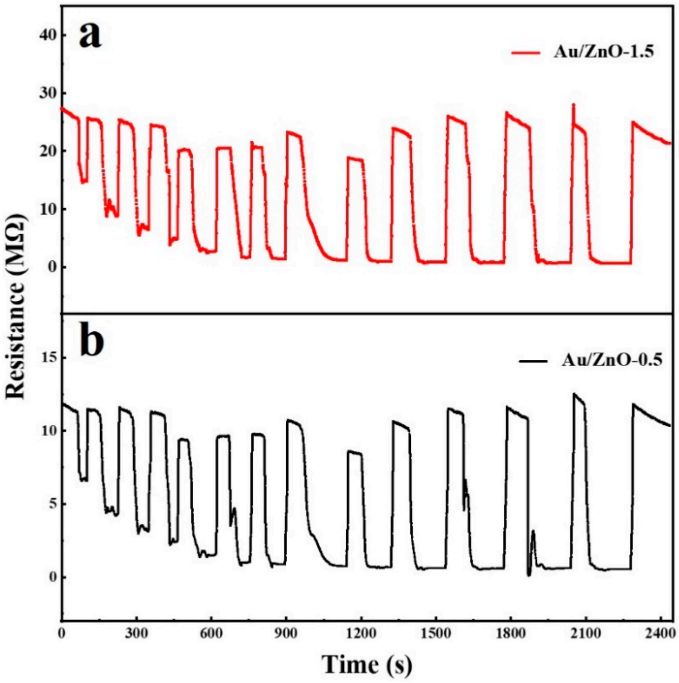

Figure S4. Dynamic response graph of (**a**) Au/ZnO-1.5 and (**b**) Au/ZnO-0.5 samples to ethanol (1-100 ppm).

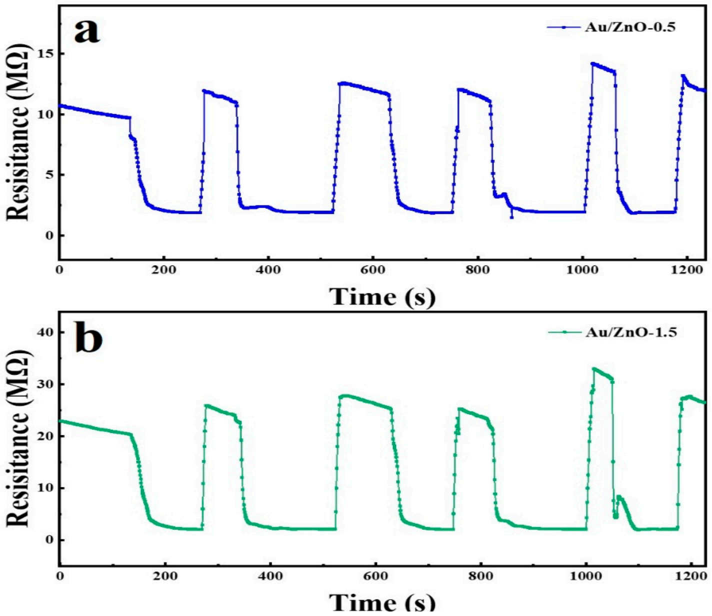

Figure S5. 5-cycle dynamic response-recovery curve of (**a**) Au/ZnO-0.5 and (**b**) Au/ZnO-1.5 samples to ethanol (100 ppm).
